# A Shortage in the Number of Nurses—A Case Study from a Selected Region in the Czech Republic and International Context

**DOI:** 10.3390/healthcare8020152

**Published:** 2020-06-02

**Authors:** Petra Maresova, Miroslav Prochazka, Sabina Barakovic, Jasmina Baraković Husić, Kamil Kuca

**Affiliations:** 1 Faculty of Informatics and Management, University of Hradec Kralove, 50003 Hradec Kralove 3, Czech Republic; petra.maresova@uhk.cz (P.M.); prochazka@zhkhk.cz (M.P.); 2 Faculty of Transport and Communications, University of Sarajevo, Zmaja od Bosne 8, 71000 Sarajevo, Bosnia and Herzegovina; sabina.barakovic@fsk.unsa.ba; 3 Faculty of Electrical Engineering, University of Sarajevo, Zmaja od Bosne bb, 71000 Sarajevo, Bosnia and Herzegovina; jbarakovic@etf.unsa.ba

**Keywords:** nurses, shortage, qualitative study, case study

## Abstract

A lack of nurses in the Czech Republic is an issue that has been under discussion for several years. The aim of this paper is to analyze the lack and need of general nurses and midwives in the Hradec Kralove region where the shortage is higher than the national average. The used methods are quantitative research and structured interviews, to determine the number of nurses in healthcare institutions. The study uses data obtained from publicly available sources, i.e., Czech Statistical Office (CSO) and the National Institute of Education (NIE). The shortage of nurses in the Hradec Kralove region can be expected by 2030 to be in the range between 647.6 and 667.1 nurses while maintaining the existing conditions, that is, five times more than at present. In addition to the commonly considered measures that appear in the country’s strategies—such as improving the quality of conditions during studies and during employment, specifying or adjusting the role and competency of nurses and midwives in the healthcare system, or unifying employment standards— a focus on promoting the nursing profession can be recommended. Schools and ministries should be encouraged to focus on and invest in the promotion of this profession, so as to play a key role in recruiting new students for the nursing field of study at a time when the nursing profession is perceived positively, as an embodiment of solidarity and selflessness.

## 1. Introduction

Although the role of nurses for the elderly care is crucial in super-aged societies, many countries, either developed or less developed, have faced a lack of long-term care workforce [1,2,3,4,5]. For example, a recent national study predicted a shortage of 36,000 nurses by 2030 in the UK [6], and Duvall and Andrews (2010) [7] indicated a significant decrease in the number of nursing graduates in the USA by 2020. Studies explain various factors of this projected shortage of workforce in nurses, such as physical demands, emotional burnout, job dissatisfaction, lack of commitment to their job, limited educational institutions to nurture current and future workforce, labor market changes, and managerial problems at the workplace that lead to high and early worker quit rates [7,8,9]. According to the International Labor Organization (ILO), the shortage of quality long-term care for seniors in central and Eastern Europe also becomes evident; in addition, many people leave these regions to supplement the shortage of long-term caregivers in Western Europe. The share of public expenditure on long-term care in the Gross Domestic Product (GDP) of countries in central and Eastern Europe is two times less than in the Organization for Economic Co-operation and Development (OECD) countries. This expenditure represents 0.7% of GDP in the Czech Republic, 0.74% in Poland, and 0.53% in Serbia, in contrast to 1.7% in the OECD countries (ILO, 2017). Workforce shortage, in turn, results in insufficient care services for the patients in need that diminishes their health and quality of life, and also increases the social and economic burden as a long-term perspective. Therefore, the inadequacy of such a supply must be high on the agenda within federal and private health care sectors aimed at aging population.

In the Czech Republic, there appears to be evidence to the increased shortages of the nurses. According to [10], for example, approximately 19,000 nurses are expected to leave their jobs by 2025, mostly due to the retirement age. Workforce analysis suggests that when considering the changes in the demography and economy in the Czech society, approximately 25,000 additional employees should be recruited to the current nursing workforce up until 2025 to fulfil an adequate number of nurses who would provide health care services in the General Nursing and Midwifery professions [11]. The Ministry of Labor and Social Affairs in the Czech Republic also ventilates a lack of nurses indicating the urgent need for the graduates in the labor market. 

This is a problem that is being addressed across developed countries, as well as in many emerging economies. Strategies exist and are being prepared in many countries, both in terms of the education system and in terms of trying to attract graduates from abroad to come to work in the country.

The aim of this paper is two-fold: First, to characterize the situation concerning the shortage of nurses in a selected region of the Czech Republic, and second, to assess the situation in the broader context of the whole country. Furthermore, this paper presents an analysis of various strategies and government policies in the Czech Republic and in other countries in order to offer a possible solution of this problem in the Czech Republic.

The paper has been organized in the following way: After the Introduction section, the used methodology, which includes the quantitative and qualitative research method, is described in Section 2. Section 3 analyses the need for nurses in the considered region, while Section 4 covers the policies adopted in the Czech Republic and abroad. Discussion of the results is given in Section 5, while Section 6 concludes the paper.

## 2. Methodology

### 2.1. Selection of the Region

Before going into more detail regarding the study research approaches, it must be stated that this study clearly addresses the shortage of the nurses in targeted regions and the Czech Republic, and will serve as a basis for political and educational initiatives to create a workforce aimed at helping retain an adequate supply of health care professionals. Additionally, it will help in the development of a young workforce of nurses within a long-term care policy for the elderly population. Implementations in a regional context can be better promoted and then expanded across the different regions in Czech Republic. 

The Hradec Kralove region can be considered as a relevant and representative example of the situation in the Czech Republic, which has the third largest number of nurses per 1000 inhabitants. Table 1 presents an overview of the numbers of full-time equivalent nurses and midwives per 1000 inhabitants in the individual regions in the Czech Republic as of 2017.

### 2.2. Data Collection

This study has been conducted by combining the quantitative research, which consists of the collection and analysis of publicly available data, and qualitative research which is based on the conducted interview.

#### 2.2.1. Quantitative Research—Data Collection in Hradec Kralove Region

The quantitative research in this study is based on data obtained from publicly available relevant sources, including the following: Integrated Health Information Systems Czech Republic (IHIS CR), Czech Statistical Office (CSO), Healthcare Holding of the Hradec Kralove Region, Hradec Kralove University Hospital, Hradec Kralove Labor Office, Charles University Medical Faculty, Regional Office of the Hradec Kralove Region, and Department of Social Affairs and Healthcare. 

The data was collected and monitored for the category of paramedics, general nurses, and midwives with specialization. Paramedics can be qualified to perform a medical profession without professional supervision, after acquiring professional competence specified in Act No. 96/2004 Coll. The general nurses and midwives’ group with a specialization have a total of 13 different detailed occupations, according to the 5th level of the ISCO-08 classification. The biggest ones are the following [10,12]:

- Other general nurses with specialization (ISCO 22219),
- Intensive care nurses (including pediatrics and neonatology) (ISCO 22212),
- Care Nurses in the internal fields (ISCO 22215),
- Pediatric nurses (ISCO 22214),
- Station nurses (except nurses in the midwifery field) (ISCO 22211),
- Nurses for care in surgical fields (ISCO 22216),
- Care Nurses in psychiatric fields (ISCO 22217),
- Perioperative Care Nurses (ISCO 22213),
- Other Midwives with specialization (ISCO 22229),
- Intensive Care Midwives (ISCO 22222).

The study combines in one group general nurses and midwives, which refers to the categories of General Nurse §5 and Pediatric Nurse §5a under senior supervision and without senior supervision.

For the evaluation of statistical surveys, the results are divided into four categories according to the type of healthcare provided, namely: healthcare in general, emergency inpatient care, other inpatient care (follow-up and long-term care), and outpatient care [13]. These healthcare services are provided in relation to medical facilities. For the purposes of the Hradec Kralove region case study, the analyzed data are related to the following: hospitals (inpatient and outpatient), professional medical institutes, spa treatment centers, separate outpatient facilities, special medical facilities, and others (pharmacy care facility, public health protection authorities).

The most recent freely available documents containing comprehensive data are as of 2018, which includes data from 2017, which is the reference point for national as well as regional data.

#### 2.2.2. Qualitative Research—Interviews in Hradec Kralove Region

When it comes to the qualitative approach in this study it has been conducted by utilizing the interview method. This type of data collection is time consuming, therefore data is collected from a smaller sample, but the benefit is that this approach offers richer information and a deeper insight into the phenomenon under study. In the concrete case, our data collected from the publicly available sources has been amended by the information obtained during interviews which allowed us to gain deeper understanding of the issues related to nurse shortage in the relevant region. This also explains the main purpose of conducting the interviews—gaining deeper insight and understanding of the topic and helping in adequate interpretation of the data collected in the quantitative part of the study.

The qualitative data was obtained by means of unstructured face-to-face interviews with relevant representatives of relevant medical facilities in the Hradec Kralove region, with the purpose of obtaining current data concerning the nurse shortage. Unstructured interviews can be referred to as “depth” or “in depth” interviews with very little structure. This means that the interviewer does not need to have prepared questionnaire, but he/she can frame the interview questions based on the interviewee and his/her previous response which allows the discussion to cover areas in great detail. In other words, this type of qualitative research involves the researcher wanting to know or finding out more about a specific topic without there being a structure or a preconceived plan or expectation as to how the topic will be handled. 

Interviews were conducted with the director of the Healthcare Holding of the Hradec Kralove Region, a representative of the Hradec Kralove University Hospital, and the director of the Hradec Kralove Labor Office. These persons were characterized as relevant personnel from the relevant medical facilities in the subject region. The subject of discussion was the question of how to obtain comprehensive data on the shortage of nurses, that is, the overlap of graduates registered with the Labor Office and the information concerning the need for nurses in individual hospitals, and these points were discussed with the director of the Healthcare Holding to validate the relevance of obtained data. This concerns the interpretation of the obtained data regarding the number of graduates, the assessment of value in relation to the possibility of entering the labor market, and pointing out insights such as the fact that many graduates already work in the field. 

The interviews were led by one or two experienced evaluation team members. Each interview was noted down in a prepared record sheet. The interviewees responded willingly and honestly, and in many cases, they supported their statements with many additional facts and information. Additionally, it is important to stress that the interview was based on the principles of intervention logic [14]. 

## 3. Need for Nurses in the Hradec Kralove Region

### 3.1. Situation in the Czech Republic

Since 2004 (when the methodology was changed and nurses were divided into individual categories), the number of nurses and midwives in the Czech Republic has increased by 1.3%—which is significantly less than in the case of doctors. While in 2004 there were 83,241 nurses and midwives in the Czech Republic, in 2016 it was 84,320 (i.e., 125.5 people per nurse/assistant). As to the registered number of employees, this number decreased by 1%, and on the contrary, the number of contract workers increased significantly—more than threefold. The great decline was in the number of pediatric nurses, since their number decreased by almost 38%.

**Figure 1:**
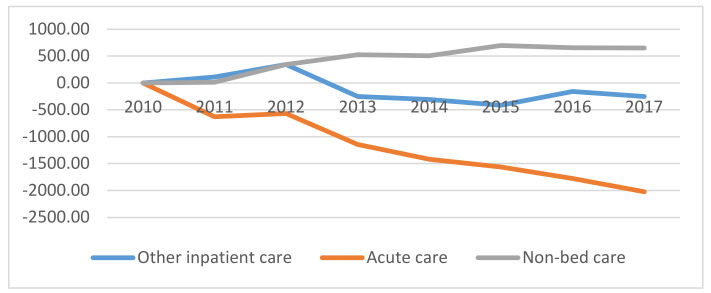
Development of the full-time equivalents of general nurses from 2010 in the Czech Republic.

The overall development of the employment of general nurses and midwives since 2010 is shown in Figure 1. In comparison to 2010, there was a decrease in the total number of full-time equivalents for emergency and other inpatient care, and an increase in the number of full-time equivalents for outpatient care. Nurse employment in emergency care dropped by 2024 full-time equivalents, in other inpatient care by 251 full-time equivalents, and in outpatient care there was an increase by 652 full-time equivalents. Compared to 2016, in 2017 there was a decrease in the full-time equivalents of general nurses and midwives in emergency care by 0.50%, in other inpatient care there was a decrease by 1.53%, and there were no major changes in outpatient care in 2017. Figure 1 illustrates the development of the full-time equivalents of general nurses and midwives, including contractual agreements, cumulatively from 2010.

The development in the number of nurses in the Czech Republic in terms of full-time equivalents is presented in Table 2. The data show a decrease in the number of employees, which reflects the trend in the number of beds decreasing or, in some cases, wards closing owing to the shortage of nurses and midwives [15]. 

### 3.2. Situation in Hradec Kralove Region

The lack and need of general nurses and midwives in the Hradec Kralove Region is higher than the national average. Specific values as well as the outlook for 2030 are characterized by the following: demographic development of the population in the Hradec Kralove Region, number of medical workers, paramedics, without professional supervision and nurses and midwives without professional supervision in individual establishments, a demographic structure of nurses and number of students and graduates in the field. 

The Hradec Kralove region has an area of 4759 km^2^ and at the end of 2015, it had 551,421 inhabitants, representing 5.2% of the Czech Republic’s population. Based on an average age of 42 years, it can be stated that the Hradec Kralove region has the oldest inhabitants of all regions, along with Zlín Region and the Capital City of Prague. The largest municipality and at the same time the seat of the region is the city of Hradec Kralove with 92,891 inhabitants, the territory of the region is divided into five districts [17]. In the Hradec Kralove region there has been an approximately 20% increase in people aged 65+ in the years between 2002 and 2015 [18]. The number of children under the age of 15 in the Hradec Kralove region will be a fifth lower by 2050. The economically active part of the population aged 15–64 years will continue to decline to three quarters of the current state in 2050. The share of this group will drop from the current two-thirds to 55%. By the middle of the century, the proportion of 65 years and older people in the region’s population will increase to almost a third. The average age of the population will grow in the region by seven years, from the current 41.8 years to 48.9 years [6]. In 2050, the age index will more than double (Table 3). 

The current and presumed future population development in the Hradec Kralove Region, taking into account the state of the populations’ health in the region, points to the following trends [19]:

- Prolonged lifetime, population aging. This is accompanied by an increase in the proportion of older age groups, therefore an increase in illnesses;
- A change in the structure of diseases from acute to chronic is envisaged, the chronic ones mainly includes [19]:
  - Tumor diseases;
  - Circulatory system diseases;
  - Musculoskeletal disorders;
  - Metabolic disorders (diabetes, obesity);
  - Chronic obstructive lung disease;
  - Visual disturbances;
  - Hearing disorders;
  - Kidney disease;
  - An increase in the number of nervous system diseases (dementia).

From the national values [17], it follows that the hospitalization within the population is highly dependent on the population’s age structure. In general, illnesses in both genders start to gradually increase with increasing age, men from about 50 years of age and women from around 60 years of age. 

### 3.3. Development in the Number of Health Workers in the Hradec Karlovac Region

In the Hradec Kralove region, the number of health workers is growing in separate outpatient facilities and in special health facilities. The numbers in the monitored period are decreasing in spa clinics and other facilities. In terms of data availability, the method of data collection, registration, and presentation of data to the public changed during the monitored period. Based on freely available sources, the current comparable value for nurses was given (with respect to efforts at minimizing bias) only in total for the year 2018 (Table 4).

### 3.4. Age Groups of Nurses in Facilities in the Hradec Kralove Region

In total, the number of health care providers in the Hradec Kralove Region is 1499, of which 24 bed care providers (including one-day bed care). From the point of view of future development and assessment of the state of care by nurses, the distribution into age groups is significant. Most people are in the 40–44 age group. The lowest number is in healthcare facilities in the 60+ category. From the 50+ age group, there are all those who will be the group at a pensionable age by 2030. In healthcare facilities, there are almost 27% of the total number of workers, in social services it is over 52%. In the lowest age category up to 29 years, which includes graduates from the last seven years, there are 11.8% of nurses in all facilities. The nationwide figure of the number of graduates is higher compared to Hradec Kralove region (HKR), i.e., 14% of fresh graduates over the last five years [11]. When comparing this data, it is clear that graduates in the Hradec Kralove region are less than the national average (Table 5).

### 3.5. Graduates and Students in the Hradec Kralove Region—General Nurses and Midwives

Secondary Medical School Hradec Kralove and Trutnov

Detailed information on HKR graduates is available at Secondary Medical School Hradec Kralove and in Trutnov (Table 6). Over the monitored nine school years, a total of 556 pupils enrolled and 325 completed their studies. There were 211 graduates in the health service, with a smaller number of students registered either outside of the healthcare field, abroad, or studying at university, on maternity leave (3 in total), in the Labor Office registry (4), and in one case no further information is available. 

Therefore, the annual average value is 40.6 graduates from universities in the region, of which 26.4 will start work in the healthcare field. (It is not possible to specify precisely how many of them will find work outside the region.)

Faculty of Medicine, Charles University: There are no graduates of the full-time Faculty of Medicine study, the first graduates should be in the academic year 2017/18, numbering 11 graduates, 20 graduates are expected for 2018/19 and 30 graduates in the academic year 2018/19. The expected average for 2017–2019 will be further considered by 20 graduates. Part-time study graduates are not considered, as in the majority of cases they are people already working in healthcare and complementing their qualification.

### 3.6. The Need for Nurses in the Hradec Kralove Region and Outlook to 2030

The specification of the number of required nurses in the Hradec Kralove region results from the synthesis of quantitative data from publicly available sources and the additional information from the interviews. Interviewees also verified that there was no duplication or uncovered areas regarding data. A representative of the Hradec Kralove University Hospital provided information about lack of nurses and midwives in healthcare facilities. The director of the Hradec Kralove Labor Office stated the fact that it is necessary to deal with the number of graduates in the region because graduates are often already in practice. So, it is not a completely new workforce. The director of the Healthcare Holding of the Hradec Kralove region discussed the results of the expected number of missing nurses.

The potential number of new nurses from the Hradec Kralove region is based on the average number of graduates from the university in HKR for the period 2007–2016, which is 40.6, and based on the expected number of new full-time study graduates at the Faculty of Medicine at Charles University in the period of 2017–2019, which comes to 20 graduates. 

Part-time study graduates are not included as they are people who study for additional qualification and already work. In total, it concerns 60.6 new graduates. This number assumes that all nurses will start working in their study field. However, according to data from the university in HKR, out of the average 40.6 graduates, only 26 new graduates started work (average for 2007–2016). From the demographic structure point of view in the horizon up to 2030, it can be deduced that every year on average 88.3 nurses leave health care facilities to retire.

Within the present state, there are a lack of nurses and midwives specified in 92.5 healthcare facilities (of which 6.5 is in Hradec Kralove University Hospital, 74 Hradec Kralove Healthcare Holding, 12 nurses in Trutnov).

**Figure 2:**
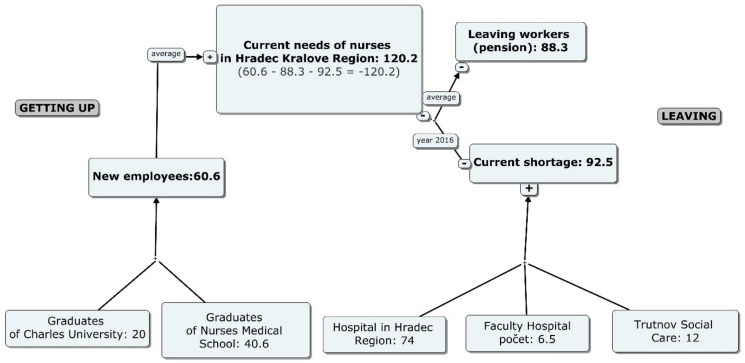
The need for nurses in Hradec Kralove region in 2016.

At this point in time, the annual difference between supply and demand is given by comparing the following: the number of graduates (for whom it is expected to start their practice in the Hradec Kralove region), the number of retired people (leaving workers) and current shortage of nurses. This is a shortage of 120.2 workers (−120.2 = 60.6–88.3–92.5). This value from another point of view is also monitored by the Labor Office, which registers 112 applicants in the position of "nurse", with the fact that not all applicants go through the register (Figure 2). 

### 3.7. Hradec Kralove Region—Outlook to 2030

Every year on the horizon for the next 10 years, we can expect the need for 88.2 nurses per year just simply to cover for retiring workers. Furthermore, on the basis of existing knowledge in the preparation of the strategy of the Hradec Kralove region in the area of social services up to 2026, an increase in the capacity of accommodation services for elderly is expected to amount to approximately 200 beds for every 3 years. This will cause a need for approximately 35 workers in the position of “general nurse” every three years, i.e., around another 12 general nurses per year (at least until 2026). Another year-on-year increase regarding the needs for healthcare personnel in social service facilities, associated with increased prescriptions of healthcare tasks, can be estimated at approximately 0.5–1% per year (i.e., around 2–4 jobs per year, 3 jobs will be considered for the next calculation).

Assuming the preservation of the current annual number of graduates (the current average number of graduates is 60.6) and accepting the hypothesis of 100% graduate admission to health and social service providers, in terms of the long-term outlook, it is possible to calculate the shortage increase as follows:

Modelling summary

- the current shortage of nurses in HKR: −92.5 to –112
- annual increase in new graduates: +60.6
- expected average annual retirement: 88.3
- expected annual need for new nurses for the social services: −15

Calculation:

(1) $$-647.6=-92.5+\left(60.6-88.3-15\right)\times 13$$

(2) $$-667.1=-112+\left(60.6-88.3-15\right)\times 13$$

In this perspective, the increased needs of the aging population are not taken into account. By 2030, a 40% increase is expected in the number of people 65+, who have a significant impact on the number of hospitalized patients and therefore, this will probably increase the need for other nurses.

## 4. Policy in the Czech Republic and Abroad

The current policies for the education of nurses in the Czech Republic are governed by the Act on Non-Medical Health Professions, i.e., Act No. 96/2004 Coll., On the Conditions for Acquiring and Recognizing Competence in Non-Medical Health Professions and Activities Related to Health Care and Amending Certain Related Acts, as amended [19]. Professional competence to practice the profession of general nurse can currently be acquired in the following ways [20]:

- Completion of at least a 3-year-long accredited bachelor’s study program in the field for the preparation of general nurses;
- Completion of at least a 3-year-long course of study at higher medical schools in the field of a certified general nurse;
- Completion of at least one year of study in the field of a certified general nurse at a higher medical school for medical workers who have acquired professional competence to perform the profession of general nurse, paramedic, midwife, or pediatric nurse according to § 5a par. 1a) or b), for medical workers admitted to higher than the first year of education.

The Ministry of Health of the Czech Republic confirms that the Act on Non-Medical Health Professions allows, as of September 1, 2017, some health professionals to acquire professional competence to perform the profession of general nurse in a shortened course of study—at least one year—at higher vocational schools in the field of certified general nurse. This involves health professionals who have previously completed their studies in a similar field: graduates of secondary medical schools in the field of medical assistant, midwife, paramedic, or nurse. The current policies make it possible to recognize such parts of the original study program that are identical to the current field of study for a qualified general nurse. This shortens their path to the profession of general nurse [20,21].

The Czech Republic introduced this policy in 2017 in order to deal with the shortage of nurses. This policy however has not yet sufficiently affected the needs of the healthcare system. 

According to Marc et al. (2018) [22], there is not a single identifiable cause of the shortage of nurses but rather a complex interplay of causes. Among them are factors that could not have been influenced, though they should have been foreseen: such as the demographic changes leading to the increasing number of senior citizens and the accompanying increasing demand for healthcare professionals. Other factors include poor planning, ineffective use of available resources, and inadequate recruitment leading to understaffing. The shortage of nurses is reaching critical levels both regionally and globally. Therefore, there is a need for concerted action on the government level, where the recommendation for an effective nursing policy is to inquire into social security services targeted at nurses. Steps must be taken to improve the education and employment of nurses, including an improvement in salary policies, and offering the nurses the opportunity of sustainable life-long learning.

One of the examples of such policies is provided by Poland, which released a new Strategy to Develop Nursing and Midwifery in Poland on 9 January 2018, addressing the following issues:

- Provision of healthcare services;
- Cntinuing education for nurses and midwives;
- Establishing a new medical profession to aid the nurses;
- Specifying the roles and competences of nurses in healthcare;
- Ensuring adequate employment standards;
- Improving working conditions;
- Promoting the prestige of the nursing profession [21].

It is obvious that the situation in Poland is very similar to that of the Czech Republic.

In developing economies, the solution of the problem starts with identifying the weaknesses in the system [23]. In Thailand, for example, one of the problems is a lack of workforce planning [24], which includes a non-existent communication between the educational institutions providing nursing education and the hospitals requiring qualified nurses. As a result of this poor organization, nurses have to do physician’s work on one hand and administrative work on the other hand, as along with a shortage of nurses, there is also a shortage of physicians. Furthermore, there are issues identified in a lack of qualified nursing instructors and a varying quality of nursing institutes. 

For comparison, in the United States, where the healthcare system is based on an entirely different system of financing, the American Nurses Association presses for funding for nursing institutes and improving access to nursing education. The association also calls for a greater diversity of nursing staff in order to better reflect the diversity of patients. There is also an initiative to motivate nursing students to pursue jobs in understaffed regions with the incentive of assistance with student loans [25]. In Ohio, for instance, lack of funds is the number one barrier for students who would seek to acquire a Bachelor of Science in Nursing (BSN). Therefore, by making education more accessible for prospective nurses, the number of nursing graduates will rise, and so will the quality of healthcare. 

Finally, in Australia, the focus is on increasing the retention of nurses and decreasing their turnover. This goal again requires coordinated efforts involving a number of parties, which also means that the policy makers must be aware of the needs of the country as a whole and of its individual regions. Focus is also on indigenous education and employment, including the enforcement of a culturally safe working environment. Policies to these ends include promoting collaboration between the educational sector and the healthcare sector to ensure that educational institutions produce such graduates as required in the job market, as well as promoting employment policies that offer retention incentives to nurses while at the same time allowing for flexibility of employment [26].

Not only Australia but also other countries around the world provide evidence that prioritizing retention is a successful strategy in dealing with the shortage of nurses, which furthermore saves costs and improves continuity of care. However, such retention programs are still at their early stages, and there is an “implementation gap” between empirical evidence and practical solutions [22]. 

## 5. Discussion

The obtained results for this study show that the shortage of nurses in the Hradec Kralove region is expected to be in the range of −647.6 to −667.1 nurses by 2030 if the existing conditions are maintained. These values are an estimate of the real need of the data obtained and should always be interpreted in the context of all the circumstances that affect them. At present, based on the shortage of nurses and the expected further decline in their numbers, the Hradec Králové region has decided to attract future nurses to hospitals with the incentive of scholarships in the amount of up to 80 thousand crowns. This could produce up to 70 paramedics next year. The requirement is that after the successful completion of their studies, the graduates will work in the hospital for at least three years. Students will receive scholarships from hospital funding. Students from all over the Czech Republic are eligible to apply [27]. In the school year 2018/19, the first students could apply for these scholarships. The results depend also on the extent of this activity.

Nursing shortages are not new to the healthcare industry, but are sometimes considered as a myth [23]. However, taking into account our results as well as reported policies worldwide, it is evident that the nursing shortage is a fact in the HKR, the Czech Republic, Europe, as well as in the whole world, and that it represents a great issue with a tendency to get even more complex due to population aging in the next years. This trend is the consequence of several issues such as: lack of potential educators, attrition from nursing programs, high turnover, inequitable distribution of the workforce, increase in aging population, aging workforce, nurse burnout, career and family, violence in healthcare settings, dealing with emotional responses such as anxiety, fear, pain, depression and anger on the part of nursing home residents and their families, etc. [24,25,26,27]. In view of the current situation, these problems are even more apparent. Zhang et al. [28] state that healthcare staff reached the cut-off values for mental disorder concerns on distress (20.1%), depression (20.6%), and anxiety (28.0%). Furthermore, older workers enjoyed better mental but not physical health. 

There are a number of proposed methods for contributing to the decrease of this problem such as: improving the quality of conditions during study and during employment, specifications or adjustments of the roles and competences of nurses and midwives in the healthcare system, unification of employment standards, increasing the salaries, redesigning the work environment, improving the public’s perspective, retaining the existing nursing workforce, developing a clinical specialist program, transition programs for new graduates, training for more certified nursing assistance, importing of staff from other countries, or flexible work schedules [25,29,30]. All of these approaches, together with the mentioned strategies and policies were simply not enough so far to meet this challenge.

According to the Health Education England [31], there is a need for concerted action on the government level, where the recommendation for an effective nursing policy is to inquire into social security services targeted at nurses. Steps must be taken to improve the education and employment of nurses, including an improvement in salary policies, and offering the nurses the opportunity of sustainable lifelong learning. Aboshaiqah (2016) [32] suggests addressing the shortage of nurses by boosting the prestige of the nursing profession. This should be done through educational activities, positive media presence, and also improving the conditions of nurses at their workplace, including improvements in work culture, such as promoting teamwork and a healthy working environment. Bloomfield, et al. [33] reports that the nursing students’ aspiration to work in primary healthcare is connected with older age, greater perceived value of employment conditions, and less perceived importance of workplace support. Therefore, collaborative efforts from health experts, academics, and policy makers are needed to attract nurses to primary healthcare (Bloomfield et al. 2018, [33]). Similarly, Neville, et al. [34] reports that some serious decisions are expected from health experts at community and government levels to attract undergraduate nurses to choose gerontological nursing as a career [34], since elderly care is viewed as an unattractive area by nursing students in Western countries [35,36,37]. However, nursing students from Eastern countries have neutral to slightly positive attitudes toward working with the elderly [38,39]. In addition, Xiao et al. [35] found out that collectivist culture has a more positive impact on nursing students’ attitudes toward the elderly compared with the individualist culture [35]. On the other hand, in order to keep the possession of older nurses’ experience, there is a need to modify workforce strategies taking into the account the patterns of workforce leaving and reentering, differences related to employment settings and practice areas, and working hours preference [40]. 

The European Union, according to Standing Committee of Nurses of the EU (PCN) [29], supports the mobility of nurses and promotes their right to practice their profession in a country different from that where they received their nursing education. Healthcare employers are therefore encouraged to consider applications from nurses trained in other countries equally and without bias.

However, what is missing is a multidimensional and structured approach and out-of-the-box thinking to this profession. On one hand, this can be achieved by gaining deeper understanding of needs of the persons in this profession, i.e., a personalized approach. This is a task for managers, work organizers, etc. On the other hand, this profession needs to be considered in interplay with other aspects of everyday life such as society, economy, migrations, etc. That is a task for policy makers, governments, etc. 

For example, a decrease in number of nurses could be partly solved by helping the retention of older nurses who want to continue working but feel burn-out by offering them flexible working, flexible retirement, flexible time off, etc. In other words, one should take a supportive approach and consider flexible working patterns or job design if someone is finding their current role difficult. Additionally, someone’s age should not be a reason to justify or tolerate poor performance or to insist on a higher level of performance that usual [41]. It is important to mention, that this approach with retention of the older work force is not characteristic only for this profession, but it will be a need in other everyday life segments due to aging populations.

According to the WHO, the shortage of nurses is only one part of a larger issue. Demographic changes, technological development, and new treatment possibilities mean that healthcare provision is an increasingly multidisciplinary and complex process. Therefore, healthcare professionals must adapt to new challenges, including acquiring new technical skills to make the most out of advanced technological treatment possibilities and learning to collaborate in multidisciplinary teams. Ultimately, all the parties involved in healthcare provision, be it nurses, physicians, patients, as well as educational institutions, hospitals, local and national governments, and other interested parties, need to learn new ways of cooperation to meet the needs of the present society [42].

The loss of nurses needs to be stopped by making the profession of nurses and other health care professionals more attractive professions for graduates as well as for experienced nurses who have left their field or are seriously thinking about it. Another possible solution is the recruitment of foreign personnel. There are a lot of proposals, and it is clear that a major change will not help one of them, but probably a great effort aimed at combining the above options.

Additionally, the workload and burden that nurses carry in their daily tasks can be lightened by relying on information-communication technology (ICT) solutions and the digital environment. Namely, Aslan, et al. [43] reported that nurses have a great work load, but they do not prioritize patient care, instead they spend time rather with treatment and registration. If ICT solutions are used in general for the improvement of quality of life of population [44], then they can help in conducting the tasks that nurses do, e.g., simplify the procedures, automate the paperwork, or even take care of certain patients. For example, Chang, et al. reports that nursing home staff prefer robot assistance who fit into their working process [40]. In this way we would increase the quality of life of nurses as well as their satisfaction with the work and decrease the burden and stress, which would result in improvement of the system that would contribute to better life quality of patients as well if the emerging ethical issues are addressed [45]. However, this study has some limitations. The first concern is the size of the study area and the number of nurses. This limitation then fails to describe the extent of the problem in full. It would be beneficial to have the exact number and predictions on shortage for other regions in the Czech Republic as well, but that remains as future work. The second limitation is the unavailability of complex current data in the given problem. Data are collected from sub-sources, which also causes their timeliness.

## 6. Conclusions

The aim of this paper was to analyze the situation regarding the nursing shortage in the Hradec Kralove region in the Czech Republic as well as in the whole country. Obtained results showed that the issue of missing nurses is present in this region and it is expected to be in the range of −647.6 to −667.1 nurses by 2030 if the existing conditions are maintained. This situation will probably be reflected in the whole country in similar scope. Additionally, this paper analyzes the strategies and policies not only in regards to the Czech Republic, but in the international context as well.

What can be concluded after the analysis is that this issue of nurse shortage is not a myth, and there have been numerous attempts worldwide to mitigate this challenge. However, as discussed, the proposed solutions, policies, and strategies were not particularly successful so far. What is missing is a multidimensional and structured approach and out-of-the-box thinking toward this profession and for solving this issue. This novel approach should be a package that applies combined personalized and social aspects to join currently proposed actions and solutions. An example of a partial solution would be to target this issue simultaneously from several points: schools and ministries to focus on and invest into promoting the nursing profession, existing healthcare institutions to provide flexible working environments for experienced nurses, usage of digital ICT solutions to unburden the load during daily tasks, etc. 

The importance of solving the issue of nurse shortage in the considered region, the whole Czech Republic, Europe, and the whole world as well is urgent. The alarm is real, and future research work should be devoted to finding structured and innovative approaches to deal with this matter.

## Figures and Tables

**Table 1:** Number of full-time equivalent nurses and midwives per 1000 population in all regions of the Czech Republic in 2017.

Number of Nurses Per 1000	Region in Czech Republic
11.67	Capital City of Prague
8.40	South-Moravian region
8.21	Hradec Kralove region
8.20	Olomouc region
7.88	Pilsen Region
7.88	Highlands region
7.72	Karlovy Vary Region
7.60	Moravian-Silesian Region
7.12	Usti Region
6.93	Zlín Region
6.87	Pardubice Region
6.76	South Bohemian Region
6.11	Liberec region
5.41	Central Bohemian Region

Source: [12].

**Table 2:** Development in the number of nurses and midwives, 2012–2017, with an outlook to 2030.

Year	Emergency Care	Other Inpatient Care	Outpatient Care	Total
2012	49,527	6098	28,077	83,702
2013	48,952	5883	28,256	83,090
2014	48,676	5829	28,238	82,744
2015	48,535	5721	28,432	82,688
2016	48,316	5977	28,387	82,680
2017	48,074	5886	28,386	82,345

Source: own, according to [16].

**Table 3:** Selected demographic indicators for the Hradec Kralove region (2018).

Region	Average Age			Age Index		
	2018	2031	2051	2018	2031	2051
Capital City of Prague	41.9	43.8	44.7	123.2	147	165
Central Bohemian	41.1	44.1	46.7	119.6	148	199
South Bohemian	42.5	46.1	48.8	103.0	191	246
Pilsen	42.6	45.9	48.3	127.0	189	237
Karlovy Vary	42.7	46.6	49.6	129.7	214	278
Ústí nad Labem	41.8	45.7	48.8	132.4	186	255
Liberec	41.9	45.5	47.9	120.1	178	226
Hradec Kralove	42.9	46.4	48.9	122.6	196	250
Pardubice	42.3	45.7	48.3	136.3	182	237
Highlands	42.6	46.5	49.9	126.5	203	277
South-Moravian	42.3	45.7	48.3	130.4	183	236
Olomouc	42.6	46.4	49.3	123.6	199	264
Zlín	42.9	46.9	50.3	130.2	214	290
Moravian-Silesian	42.5	46.3	49.6	134.8	201	273

Source: edited by [12].

**Table 4:** Development in the number of nurses in healthcare facilities in the Hradec Kralove region.

Year	Hospitals (Bed and Outpatient)	Professional Medical Institutes	Spa Treatment Centres	Separate Outpatient Facilities	Special Medical Facilities	Other *	Total
2000	3475.57	424.40	X	1597.98	163.40	331.20	5992.55
2001	3549.71	425.56	X	1619.61	157.83	356.55	6109.26
2002	3599.98	435.66	X	1664.90	150.64	367.99	6219.17
2003	3519.25	410.21	X	1674.24	190.81	279.23	6073.74
2004	3343.83	348.55	X	1529.95	218.88	296.99	5738.2
2005	3366.18	338.19	X	1525.92	205.28	288.14	5723.71
2006	3379.43	248.51	94.31	1509.12	196.72	257.55	5685.64
2007	3449.39	247.26	101.12	1561.55	204.69	257.10	5821.11
2008	3441.54	239.66	90.99	1606.01	211.36	247.28	5836.84
2009	3470.88	249.93	89.39	1640.38	221.01	237.86	5909.45
2010	3452.97	253.54	89.95	1635.71	238.45	232.85	5903.47
2011	3400.72	247.77	90.34	1657.38	244.43	227.01	5867.65
2012	3445.90	254.67	91.48	1668.32	250.59	221.13	5932.09
2013	3430.27	255.02	69.24	1669.85	272.02	234.80	5931.2
2018	x	x	x	x	x	x	4682.8
2030	3495.50	422.20	98.98	1583.24	156.91	341.91	4938.4

* Sum of items: Pharmacy care facility, public health protection authorities, other Source: own, according [20].

**Table 5:** Age groups of the nurses in the Hradec Kralove region (HKR).

Year	Social Services	Other Hospitals in Region	Faculty Hospital	Contributory Organisations in Healthcare	Other Outpatient	Total	Total –Layout in %
To 29	14	130	234	10	91	479	10.8
30–34	22	198	183	19	99	521	11.8
35–39	23	168	247	24	108	570	12.9
40–44	80	240	383	36	173	912	20.6
45–49	70	165	241	24	117	617	14.0
50–54	62	140	190	25	98	515	11.6
55–59	95	151	153	24	99	522	11.8
60+	72	62	81	18	55	288	6.5
Total	438	1254	1712	180	840	4424	100.0

**Table 6:** Qualified general nurse and midwives from the Secondary Medical School Hradec Kralove and Trutnov.

School Year	Number Enrolled in the Study	The Study Ended (Final Exam)	Work in Healthcare	Abroad	Outside Healthcare	Continues to Study University
2007–2008	40	18	0	0	0	0
2008–2009	40	26	0	0	0	0
2009–2010	60	28	21	5	0	2
2010–2011	60	37	29	7	1	0
2011–2012	56	62	42	0	0	5
2012–2013	60	40	33	0	2	4
2013–2014	87	50	28	0	1	1
2014–2015	95	64	58	0	2	1
2015–2016	58	x	x	x	x	x
Average	61.8	40.6	26.4	1.5	0.8	1.6
Total	556	325	211	12	6	13

Source: own, according to [20].
